# New high-fidelity terrain modeling method constrained by terrain semanteme

**DOI:** 10.1371/journal.pone.0198530

**Published:** 2018-06-07

**Authors:** Bo Zhou, Junbo Xu, Xuedong Zhang, Xuejun Liu

**Affiliations:** 1 Department of Information Engineering, HeFei University of Technology, Xuancheng 242000, China; 2 Department of Geographic Information Science, Beijing University of Civil Engineering and Architecture, Beijing 100044, China; 3 Key Laboratory of Virtual Geographic Environment, Ministry of Education, Nanjing Normal University, Nanjing 210046, China; Beijing University of Posts and Telecommunications, CHINA

## Abstract

Production of higher-fidelity digital elevation models is important; as such models are indispensable components of space data infrastructure. However, loss of terrain features is a constant problem for grid digital elevation models, although these models have already been defined in such a way that their distinct usage as data sources in terrain modeling processing is prohibited. Therefore, in this study, the novel concept-terrain semanteme is proposed to define local space terrain features, and a new process for generating grid digital elevation models based on this new concept is designed. A prototype system is programmed to test the proposed approach; the results indicate that terrain semanteme can be applied in the process of grid digital elevation model generation, and that usage of this new concept improves the digital elevation model fidelity. Moreover, the terrain semanteme technique can be applied for recovery of distorted digital elevation model regions containing terrain semantemes, with good recovery efficiency indicated by experiments.

## Introduction

A digital elevation model (DEM) provides a digital expression of terrain. With the development of new technologies [1–4], DEM applications have undergone considerable expansion [5–7]; hence, finer terrain representation is required. Terrain modeling is a DEM generation method that processes sampled data into a DEM. The grid DEM is the most common data-based model [8], being composed of square grids typically generated through a traditional process, where in a triangulated irregular network (TIN) is constructed based on sampled points and interpolated to yield grid data. Studies to improve grid DEM accuracy generally consider the interpolation algorithm [9–13] and new data structures [14, 15], which can partly mitigate native defects present in the grid structure.

DEM generation incorporates three interrelated tasks: data capture, data processing, and data visualization. The data processing interpolation algorithm is usually the core component of terrain modeling. Therefore, to improve DEM accuracy, many researchers have worked to improve the interpolation function. Examples of the resultant improvements include a new interpolation technique based on Coons patches [9, 16, 17], a new DEM generation method based on map algebra [10–12], a method based on the Gaussian Markov random field [13], and high-accuracy surface modeling [18–23], which has been proposed as a method of obtaining regular grid elevation data. In recent years, new data-sampling technology has been developed and new data processing methods have been proposed. Related techniques proposed for DEM generation include the use of point clouds provided by Light Detection and Ranging (LiDAR) [24–27], along with methods for processing synthetic aperture radar (SAR) data [28]. On the other hand, advanced DEM data structures, specifically hybrid data structures based on the grid DEM, can also be used to improve DEM accuracy. A unified data structure that presents peculiar properties in terms of both vector and raster data has been proposed [14, 29]. Further, the SCOP++ program utilizes a hybrid structure [15], and Yang et al. [30] have proposed a new model with a TIN and regular grid for multi-resolution. Although the techniques discussed above have successfully improved DEM accuracy, distortion remains, especially in the context of terrain feature loss; for example, the horizontal plane area of a grid DEM may be uneven rather than exhibiting the appropriate flatness.

The present study investigates the sources of DEM generation distortion and proposes a new terrain modeling method to solve this problem. Hence, the concept-terrain semanteme is newly proposed, so as to appropriately define local space terrain features. Then, a new DEM generation process incorporating the terrain semanteme is designed. The feasibility of this approach is confirmed using a programmed prototype system, with improved DEM fidelity and efficient recovery of distorted DEM regions being achieved.

## Proposed terrain semanteme

In this section, terrain surface composition is analyzed to determine the specific sources of DEM distortion. Then, terrain semanteme is proposed and executed to resolve this problem.

### Terrain surface composition

**Fig 1:**
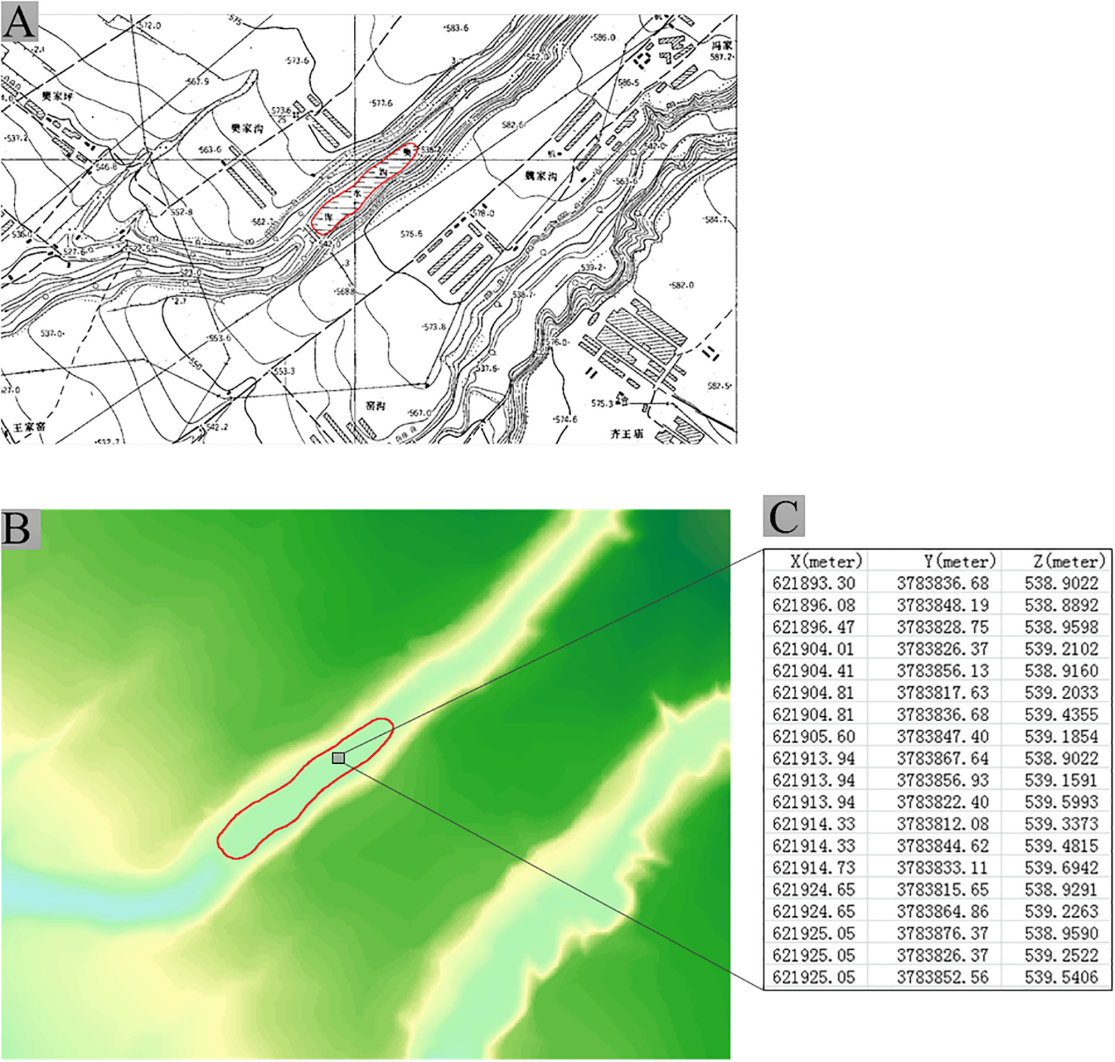
Topographic map of sample area and distorted DEM. (A) Topographic map of reservoir. (B) Grid DEM. (C) DEM elevation data.

Fig 1(A) shows a topographic map of a static-water reservoir for which the elevation data values are equivalent; however, the DEM of this reservoir (Fig 1(B)) does not yield equivalent elevation data values, as shown in Fig 1(C). The loss of equality for the reservoir elevation data generates distortions in grid DEM.

**Fig 2:**
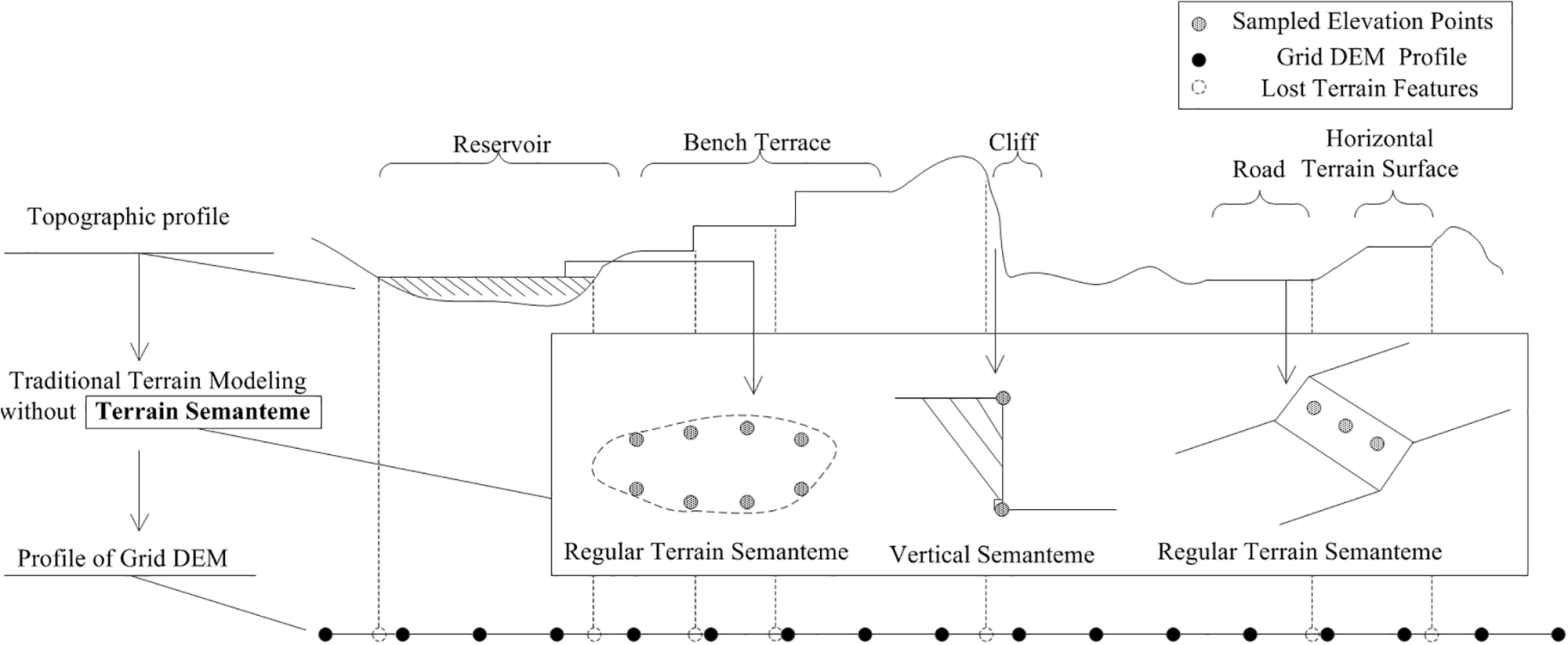
Analysis of terrain surface features that tend to generate DEM distortions.

So, application of constraints to the elevation data used in the DEM production flow can prevent the appearance of such distortions. Therefore, in this study, the terrain surface composition was analyzed to characterize and utilize such a constraint, as shown in Fig 2. This figure presents an analysis of several kinds of terrain objects that usually correspond to distorted DEMs. In general, traditional production methods cannot perfectly express certain terrain features, specifically, equivalent elevation data values (e.g., the static-water area of a reservoir, the flat regions of a bench terrace, or the surface of a road), vertical surfaces (e.g., the vertical surfaces of a bench terrace or cliff), and the inclined planes of artificial terrain. These difficulties arise because traditional terrain modeling generally recognizes a terrain surface as a type of field [31]. Traditional production methods, such as those used to obtain grid DEMs, cannot perfectly express terrain object features that can be smooth and have unconstrained interpolated data. Therefore, traditional terrain modeling retains most terrain features in the field regions, but ignores the local features of the terrain objects, thereby producing distorted DEM regions.

A terrain surface is composed of both field regions and terrain objects (Fig 2), thereby challenging the authenticity of the field-based production flow of a terrain surface. To make full use of the produced grid DEM, the terrain modeling method should be improved to retain local terrain features in the DEM production flow. In this study, the terrain semanteme is proposed as a means of describing the local terrain features of terrain objects. In addition, a new terrain modeling flow designed under the constraint of the terrain semanteme is presented, so as to obtain a new DEM that retains the local features. Moreover, new data structures are designed to express these local terrain features. This new concept can also be utilized in the recovery of distorted DEM data for a regular terrain region, as demonstrated below.

### Terrain semanteme

The terrain semanteme provides a description of the spatial features of a local terrain region and is the only semanteme of this region. Under the constraint of the terrain semanteme, the elevation model can be described as follows:

$$\{\begin{array}{ll} ElevationModel=\{M_{i}=\zeta (P_{j})|P_{j}(x_{i},y_{i},z_{i})\in D,j=1,\ldots ,n;i=1,\ldots ,m\} & \left(\text{a}\right)\\ S(P_{i})=H(P_{i}\in S_{i},S_{i}\subset D) & \left(\text{b}\right) \end{array}$$ (1)

Here, Eq (1(a)) is the description of the DEM, indicating that a terrain model (*ElevationModel* in this function) is composed of a series of slices *M*_*i*_, each of which is composed of elevation points *P*_*j*_ under rule *ζ*. Eq (1(b)) indicates that all the elevation points in semantic region *A*, which is a subset of *D*, satisfy function *S*. Therefore, Eq (1(b)) constrains the elevation data in semantic region *A*. Compared to the original DEM equation (Eq (1(a)) alone), Eq (1) incorporates an added constraint, i.e., Eq (1(b)), which is applied to the region to ensure representation of all terrain features.

Terrain semanteme can be used to describe terrain features such as those of a flat-terrain region (having equivalent elevation data values). Likewise, all similar spatial features can be described by the terrain semanteme concept.

### Characteristics of terrain semanteme

The function of the terrain semanteme can be interpreted by considering the following precondition and scale characteristic.

1. The terrain semanteme is based on the existence of terrain objects.
  The terrain semanteme is preconditioned by the existence of terrain objects. For example, the top of a mountain exists in correlation with the mountain itself; therefore, if the mountain did not exist, the terrain semanteme associated with the mountaintop would also cease to exist. Therefore, the spatial feature of the top (i.e., the terrain semanteme defined in this paper) is based on the existence the mountain, which is the main object in this example.
2. The terrain semanteme must be characterized according to a particular scale.
  The spatial feature represented by the terrain semanteme is an attribute of a terrain object in accordance with some scale. Its application is generally limited by its scale. Given that different semantemes may be associated with an object according to different scales, terrain semantemes must be characterized based on a particular scale.

### Formulization of terrain semanteme

Terrain semantemes can be broadly categorized into regular, regular-shaped, and vertical semantemes based on their descriptions, as detailed in Table 1.

**Table 1:** Formulization of the terrain semanteme.

Terrain semanteme classification	Formulization
Regular	In a semantic region of *Object*_*i*_, if *P*_*j*_(*x*_*j*_, *y*_*j*_, *H*_*j*_) ∈ *Object*_*i*_, the the point *P*_*j*_ satisfies*F*(*P*_*j*_) = 0, *F* is a plane function or a curved surface equation.
Regular-shaped	In a semantic region, some points (*P*_1_(*x*_1_, *y*_1_), *P*_2_(*x*_2_, *y*_2_), …, *P*_*n*_(*x*_*n*_, *y*_*n*_)) or (*P*_1_(*x*_1_, *y*_1_, *z*_1_), *P*_2_(*x*_2_, *y*_2_, *z*_2_), …, *P*_*n*_(*x*_*n*_, *y*_*n*_, *z*_*n*_)) satisfiy (*F*(*P*_1_, *P*_2_, …, *P*_*n*_)) = 0, *F* is a regular geometry shape, such as a circle, or a ellipse, or others.
Vertical	The Point set (*P*_1_(*x*_1_, *y*_1_), *P*_2_(*x*_2_, *y*_2_), …, *P*_*n*_(*x*_*n*_, *y*_*n*_)) contains several different elevation data values.

In a region with regular terrain semanteme, all the elevation data satisfy a given formulation and the regional semanteme expresses regularity. An example of such a case is when the horizontal and bevel terrain data satisfy a plane function, as follows:

$$\begin{array}{c} Z=aX+bY+c \end{array}$$ (2)

where *X* and *Y* are position variables; *Z* is the elevation variable; and *a*, *b*, and *c* are the parameters of this plane. A regular-shaped terrain semanteme presents points on the terrain surface that form a geometrical shape such as a circle, a square, or a triangle. This semanteme can be used to correct the region elevation in the production flow for DEM generation. A vertical semanteme is that of a vertical surface that cannot be visualized by a grid structure because of the overlapping elevation points present at one coordinate. In the present study, use of the terrain semanteme concept allowed retention of the terrain features or raw data of the vertical surface. The formulization allows modification of the terrain semanteme to obtain the DEM. Formulas can then be chosen to constrain the elevation data based on the specific terrain semanteme.

## Terrain-semanteme-constrained terrain modeling method

Following its formulization, a terrain semanteme can be used to generate a semantic DEM (S-DEM). As the terrain semanteme concept is novel, this approach has not been used previously to constrain elevation data in the terrain modeling flow. Therefore, design of a conceptual model and a terrain modeling flow constrained by terrain semanteme was required, which is described in this section.

### S-DEM conceptual model

The S-DEM data structure was designed with consideration of the DEM application and based on the grid DEM, which is the most widely used data structure. S-DEM data are stored in two data layers, namely, the grid and feature data layers. The grid data layer carries the grid structure data and is identical to the simple grid DEM, whereas the feature data layer is composed of feature lines and TINs. The S-DEM can be formulized as follows:

$$\{\begin{array}{ll} ElevationModel=U_{i-1}^{n}G_{i} & \left(\text{a}\right)\\ G_{i}=(V,F) & \left(\text{b}\right)\\ V=(V_{1},V_{2},V_{3},V_{4}) & \left(\text{c}\right)\\ \begin{array}{l} \text{F}=\{((Code_{1},(x_{1},y_{1},z_{1}),(x_{2},y_{2},z_{2}),\ldots ,(x_{m},y_{m},z_{m}));\\ \;\;\;\;\;\;\;\;\;\;\;((Code_{2},(x_{1},y_{1},z_{1}),(x_{2},y_{2},z_{2}),\ldots ,(x_{m},y_{m},z_{m}))\text{;}\\ \;\;\;\;\;\;\;\;\;\;\;\ldots \ldots \\ \;\;\;\;\;\;\;\;\;\;\;\text{((}Code_{n},(x_{1},y_{1},z_{1}),(x_{2},y_{2},z_{2}),\ldots ,(x_{m},y_{m},z_{m}));\} \end{array} & \left(\text{d}\right)\\ S=(F,A) & \left(\text{e}\right) \end{array}$$ (3)

Here, *ElevationModel* is composed of the grid *G*_*i*_; *V*_*i*_ = (*V*_1_, *V*_2_, *V*_3_, *V*_4_) corresponds to the four points of one grid; *F* represents the feature lines of one grid, which is an terrain object’s area formed by several lines; *Code* is the feature line number; *x*, *y*, and *z* are point coordinates, where the points constitute the feature line; and the semanteme *S* is composed of *F* and *A*, and *A* is the terrain semanteme attribute of the region.

**Fig 3:**
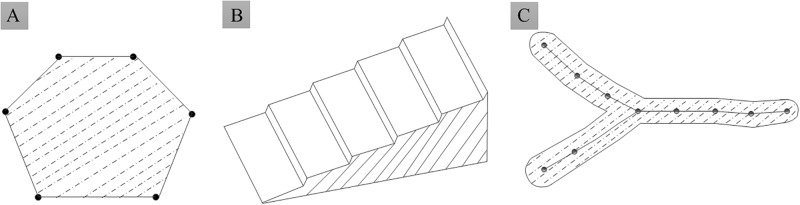
Three typical semantic region structures. (A) Atomic semanteme. (B) Combination semanteme. (C) Semanteme with a center line.

The terrain semantic region structure is classified as atomic, combination, or semantic with a center line or a center point, as shown in Fig 3(A)–3(C), respectively.

### S-DEM production flow

**Fig 4:**
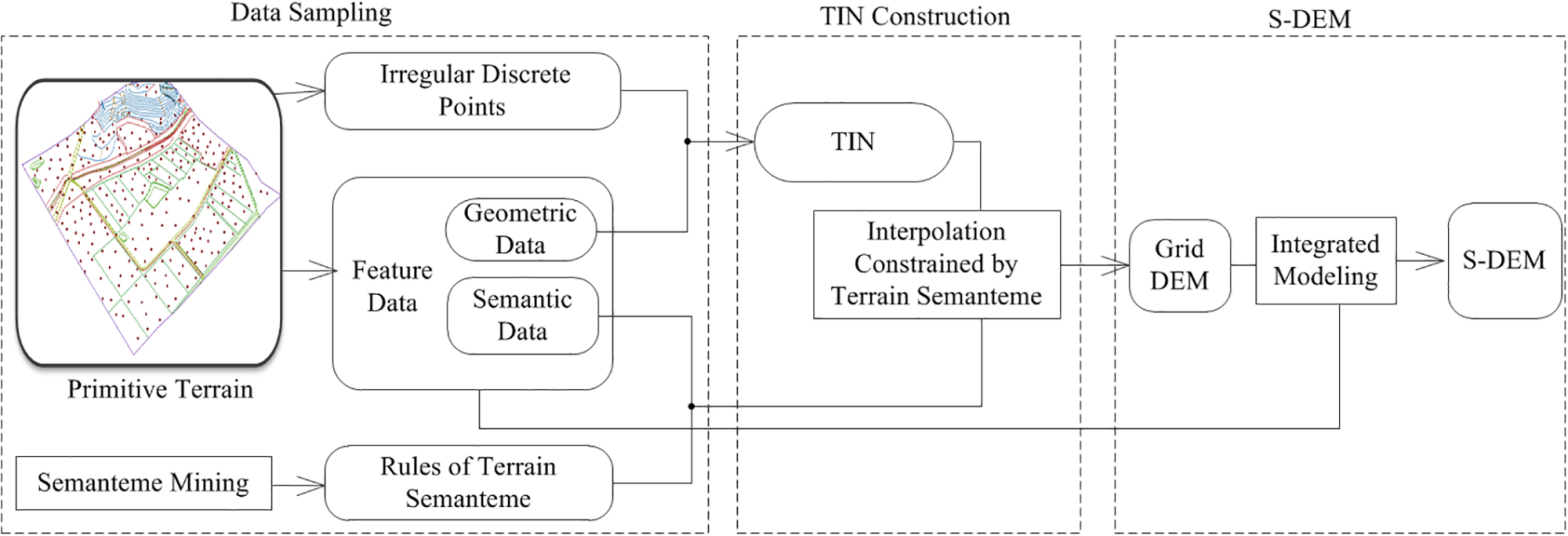
S-DEM production flow.

The S-DEM production flow (Fig 4) is as follows. First, irregularly distributed sampling point data, the feature line data, the feature surface data and the semantic attributes of the terrain objects are collected. Second, the distributed point data and feature line data are employed for TIN construction and interpolation to generate a grid data layer. Third, the S-DEM is produced, wherein the data of the semantic region are embedded into the grid data layer to complete the expression of the terrain in a three-dimensional (3D) environment.

#### Data capture

Apart from the routine terrain data, S-DEM production requires collection of semantic data, which include (1) the data of semantic regions, for example, the border lines and attributes of a natural static water region, and (2) the special terrain surface data, for example, the vertical surface data.

Following data capture, the data are detected by gross error, which involves (1) integrity detection, which is applied to a semantic region that encompasses both semantic data (*A* in Eq (3(e))) and feature data (*F* in Eq (3(b))); and (2) uniformity detection, wherein the terrain semanteme for the area must agree with the sampled data for that terrain. Otherwise, the data requires further correction.

#### Data processing

**Fig 5:**
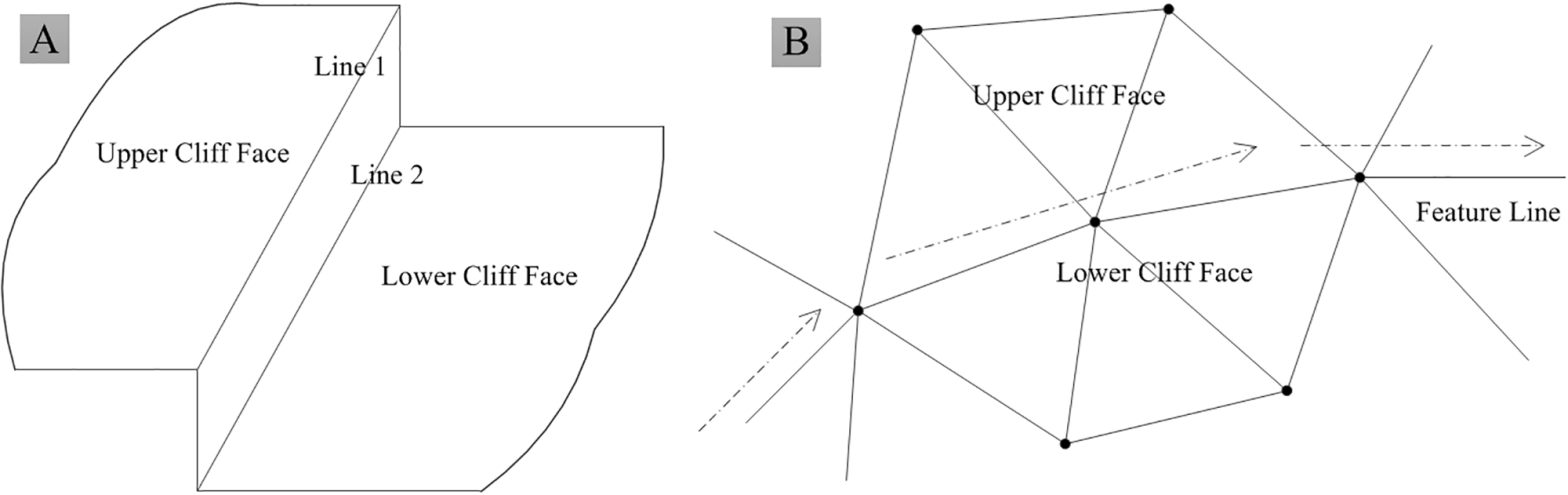
Geometrical structure of vertical plane. (A) Cliff structure. (B) Feature-line direction in TIN.

Following data sampling and detection, a constrained Delaunay TIN (CDT) is constructed using irregularly distributed points and feature data. We considered the case shown in Fig 5 as a validation experiment. In that case, flat triangles with three equally elevated points were conventionally rebuilt such that the points on the terrain semantic regions followed the terrain semanteme. In addition, the vertical surface shown in that figure was incorporated in the construction of the CDT in a two-dimensional (2D) environment, in the form of a feature line.

In this method, following the construction of CDT, the semantic regions are interpolated in accordance with the formulas presented in Table 1. The interpolation of these triangles is performed separately. For the case shown in Fig 5, the arrow directions in Fig 5(B) indicate the feature line direction in the 2D environment. The areas above and below the feature line represent the upper and lower cliff faces, respectively. Following the interpolation of this region, the succeeding interpolating points must be judged with respect to its position on either side of the feature line. Interpolation calculations for points on the side corresponding to the upper cliff face must employ upper-side terrain elevation data, and vice versa. Note that, in Fig 5(A), Lines 1 and 2 correspond to the upper-face and lower-face data, respectively.

#### S-DEM generation

**Fig 6:**
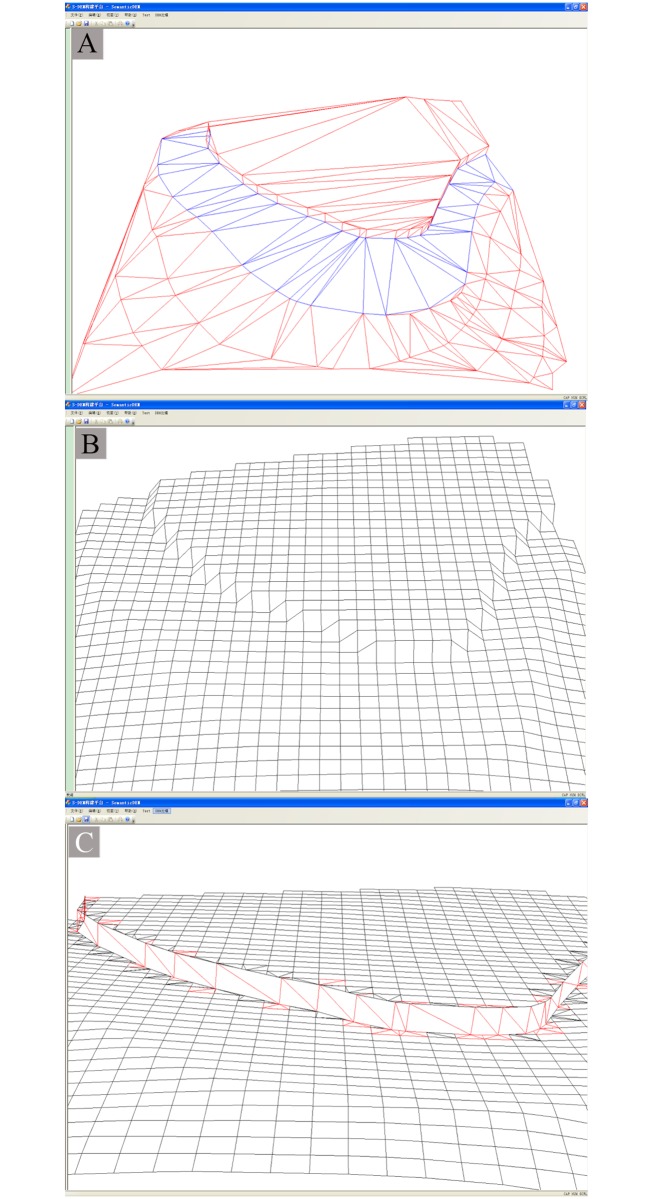
Vertical plane of Fig 5 as represented by prototype system. (A) TIN. (B) Grid DEM. (C) S-DEM of vertical surface.

Following generation of the grid data, the feature data are embedded into the grid structure to visualize the S-DEM in a 3D environment. The S-DEM embedding is divided into two categories, specifically, feature line embedding and triangulation, and vertical surface embedding. Saved data on the vertical surface are recovered following the feature line embedding. This is shown in Fig 6, for the validation experiment conducted in this study.

### S-DEM prototype system

In this study, a prototype system was programmed in Microsoft Visual Studio 2010 to simulate the S-DEM production flow. Three stages were implemented: data input, data processing, and data output. Data input involved the input of irregular discrete points, feature data, and semantic data. Data processing involved the terrain-semanteme-constrained interpolation and feature data embedding. Lastly, the data output stage involved visualization and files output.

The interface of the prototype system is presented in Fig 6. First, the TIN of the original data is constructed. In the example considered in the validation experiment (Fig 5), these data includes the data for the vertical surface, as apparent for the TIN shown in Fig 6(A). Second, the grid DEM is interpolated from the TIN, although the grid DEM inevitably loses some terrain features during interpolation. In the case of the validation experiment, the vertical surface is lost by the grid DEM, as shown in Fig 6(B). Lastly, the original feature data are embedded into the grid data to obtain the S-DEM, with terrain features being recovered. The recovered terrain features are then embedded into the grid structure in the form of TIN. In the validation experiment, the vertical surface was recovered, as apparent from Fig 6(C).

### Experimental results

In the experiment performed in this study, the S-DEM retained the terrain region feature and visualized the vertical surface of Fig 5 in a 3D environment, as shown in Fig 6. The S-DEM improved the fidelity of the terrain model. These results were influenced by the semantic data, which eradicated any discrepancy between the terrain model and terrain object attributes.

Furthermore, note that the S-DEM is based on the grid structure and feature data. The feature data retains the terrain features but are not beneficial for terrain analysis, because of the complex TIN structure. The presence of terrain objects increases the difficulty of DEM application to terrain analysis, for example, in the case of flood analysis, where a river may spread to other terrain objects. In such scenarios, the DEM must represent the topological relationship between rivers or other objects. In such cases, high-accuracy DEM may be non-conducive to terrain analysis. Related research involving the S-DEM must be conducted for further clarification of this issue.

## DEM data recovery based on terrain semanteme

The terrain semanteme concept is not limited to DEM data production under the constraint of the elevation data for a given area. In fact, it can also be employed for data recovery in a distorted region that presents terrain semantemes.

### Distorted DEM recovery method

Fig 1 shows the elevation data of a static water region, which corresponds to a regular terrain semanteme. Although the elevation data values must be equal, the listed DEM data are inequivalent (Fig 1). According to the formulization of the regular terrain semanteme (Table 1), the DEM of a water region must satisfy the relation

$$\begin{array}{c} Z=aX+bY+c \end{array}$$ (4)

If the values of *a*, *b*, and *c* are determined, the distorted DEM can be recovered using Eq (4).

In addition, the distorted grid DEM is a point set *V*_*i*_, such that *V*_*i*_ = {(*x*_*i*_, *y*_*i*_, *z*_*i*_), *i* = 1, 2, 3, …, *n*}. According to the least squares(LS) principle, the optimal values of *a*, *b*, and *c* can be obtained following attainment of the minimum value of *S*, as follows:

$$\begin{array}{c} S=\sum_{i=0}^{n}{\left(ax_{i}+by_{i}+c-z_{i}\right)}^{2} \end{array}$$ (5)

The minimum value of *S* can be determined if the partial derivatives of *S* in relation to *a*, *b*, and *c* are zero, i.e.,

$$\begin{array}{c} \frac{\partial S}{\partial a}=0,\frac{\partial S}{\partial b}=0,\frac{\partial S}{\partial c}=0 \end{array}$$ (6)

Eq (6) can be arranged to generate the following set of linear equations:

$$\begin{array}{c} \left\{ \begin{array}{c} \sum 2\left(ax_{i}+by_{i}+c-z_{i}\right)x_{i}=0\\ \sum 2\left(ax_{i}+by_{i}+c-z_{i}\right)y_{i}=0\\ \sum 2(ax_{i}+by_{i}+c-z_{i})=0 \end{array}\right. \end{array}$$ (7)

The plane constrained by the terrain semanteme can then be obtained based on the solutions of the equations presented in Eq (7).

### Verification and analysis of distorted DEM recovery

**Fig 7:**
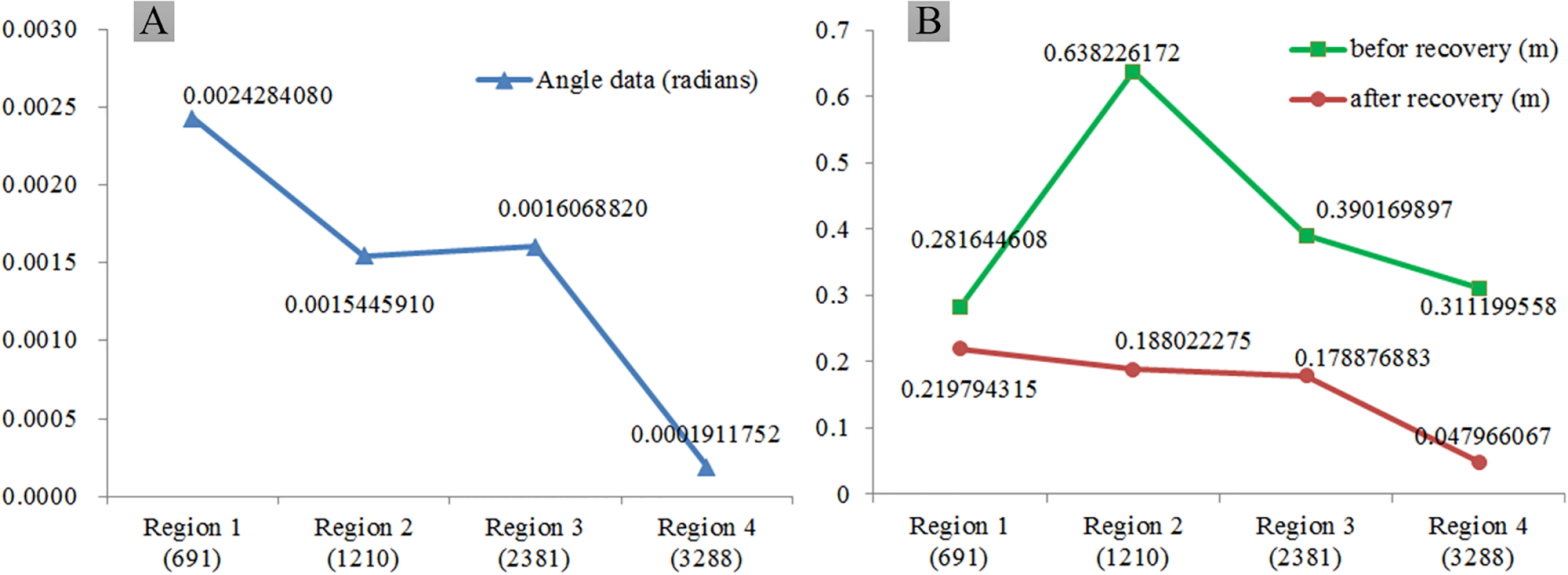
Experimental results showing DEM recovery reliability and accuracy for four sample regions. (A) Angle between recovered plane and horizontal (rad). (B) RMSEs of elevation before and after recovery (m).

Four reservoirs with distorted DEMs were selected to verify the efficacy of the terrain semanteme in recovering the distorted DEM. These four reservoirs contained 691, 1210, 2381, and 3288 grid points, respectively. After recovery, a recovered plane was obtained, and the angle between the plane and horizontal was taken to evaluate the recovery reliability (Fig 7(A)). The recovered DEM was determined according to Eq (3), and the root mean square errors (RMSEs) of the elevation before and after recovery were used to compare the data accuracy of the distorted and restored DEMs (Fig 7(B)).

Obvious distortions in the elevations of the semantic regions are apparent in Fig 7 (before recovery), indicating that the DEM cannot express a planar attribute for static water. However, following recovery, the attributes of the terrain objects in the region were obtained. Note that an increased number of grid points corresponded to increased accuracy for the DEM recovery under the terrain semanteme constraint.

Based on these results, it is feasible to recover the data of a distorted DEM using the terrain semanteme concept, and highly accurate DEM data can be obtained with sufficient sampling points.

Actually, the error on the semantic terrain region of distorted DEMs appeared randomly and presented normal distribution in the interpolation stage. The LS is a good and normal method to get an optimum value from the data with random errors. On the other hand, in other similar applications, Random Sample Consensus (RANSAC) [32] is a feasible method to get an available answer. The data of distorted DEM does have error but no wrong data which the RANSAC is good at dealing with. So, considering the complexity, the LS is chosen to get an available value of distorted DEM region in this paper.

## Conclusions

Terrain is composed of both field regions and terrain objects, and conventional DEMs often exhibit distortion due to loss of terrain features. To overcome this problem, the terrain semanteme concept was proposed in this study. Based on this concept, a new terrain modeling process and new data structures were designed to visualize a semantic DEM (S-DEM) in a 3D environment using a prototype system. Improvements in DEM fidelity were observed in experiments using the developed technique. Hence, the following conclusions were obtained:

1. Formulization of the terrain semanteme can constrain the elevation data of the semantic area during terrain modeling, thereby ensuring a correlation between the S-DEM and terrain semanteme as well as eradicating distortions in the semantic region. The use of semantic data as the DEM data source was validated; hence, the GIS data source has been broadened.
2. Terrain semanteme can be used as a data source in terrain modeling, as well as in the recovery of distorted regions of grid DEMs having terrain semanteme. In this study, recovered DEM data that were closely related to the original terrain surface were obtained. Superior results were obtained with an increased number of sampled DEM points.
3. Essentially, the terrain objects were already constructed when the terrain semanteme was expressed in the programming of the prototype system; this correlates with the concept that the terrain semanteme is essentially a representation of the terrain objects. Moreover, the characteristics of the terrain objects match well with the inheritance system of a specific object in object-oriented programming, which aided development of the terrain semanteme.

In the next research stage, considering the application of terrain analysis, the topological data can be used to connect the terrain semantic regions. So, the terrain analysis based on S-DEM can be feasible. Theoretically, this kind terrain analysis based on different semantic regions can avert defects of gird DEM and get a good result.

## Supporting information

S1 File This file includes four distorted DEMs belonging to four reservoirs respectively, these data were used to recover DEM data and the efficiency had been analyzed in Fig 7. (XLS)
